# Milton’s Blindness

**Published:** 1864-01

**Authors:** 


### MILTON'S BLINDNESS.

The following is published in the recent Oxford edition of Milton's works, as from his pen; it is certainly the production of Mrs. Howell, of Philadelphia.

I am old and blind!
Men point at me as smitten by God's frown;
Afflicted and deserted of my kind;
Yet I am not cast down.

I am weak, yet strong;
I murmur not that I no longer see;
Poor, old, and helpless, I the more belong,
Father supreme! to thee.

O merciful One!
When men are farthest then thou art most near;
When friends pass by me, and my weakness shun,
Thy chariot I hear.

Thy glorious face
Is leaning toward me; and its holy light
Shines in upon my lonely dwelling-place,
And there is no more night.

On my bended knee
I recognize thy purpose clearly shown:
My vision thou hast dimmed, that I may see
Thyself—thyself alone.

I have naught to fear;
This darkness is the shadow of thy wing;
Beneath it I am almost sacred—here
Can come no evil thing.

Oh! I seem to stand
Trembling, where foot of mortal ne'er hath been,
Wrapped in the radiance of thy sinless land,
Which eye hath never seen.

Visions come and go:
Shapes of resplendent beauty round me throng;
From angel-lips I seem to hear the flow
Of soft and holy song.

Is it nothing now,
When heaven is opening on my sightless eyes?
When airs from paradise refresh my brow
The earth in darkness lies.

In a purer clime
My being fills with rapture—waves of thought
Roll in upon my spirit—strains sublime
Break over me unsought.

Give me now my lyre!
I feel the stirrings of a gift divine
Within my bosom glows unearthly fire
Lit by no skill of mine.

